# Facilitators and barriers to latent tuberculosis infection treatment among primary healthcare workers in Malaysia: a qualitative study

**DOI:** 10.1186/s12913-023-09937-z

**Published:** 2023-08-29

**Authors:** Siti Nur Farhana H., Anusha Manoharan, Wen Ming Koh, Manimaran K., Ee Ming Khoo

**Affiliations:** 1 Institute for Health Behavioural Research, National Institutes of Health, Ministry of Health Malaysia, Block B3, Kompleks NIH, No 1, Jalan Setia Murni U13/52, Seksyen U13, Setia Alam, Shah Alam, Selangor 40170 Malaysia; 2 Bandar Botanic Health Clinic, Bandar Botanic, Klang, Selangor 42000 Malaysia; 3 Rawang Health Clinic, Jalan Rawang Perdana, Taman Rawang Perdana, Rawang, Selangor 48000 Malaysia; 4 Department of Primary Care Medicine, Faculty of Medicine, Universiti Malaya, Kuala Lumpur, 50603 Malaysia

**Keywords:** Healthcare workers, Latent TB infection, Malaysia, Primary care, Qualitative study

## Abstract

**Background:**

Healthcare workers (HCWs) have an increased risk of active and latent tuberculosis infection (LTBI) compared to the general population. Despite existing guidelines on the prevention and management of LTBI, little is known about why HCWs who tested positive for LTBI refuse treatment. This qualitative study sought to explore the facilitators and barriers to LBTI treatment uptake among primary HCWs in Malaysia.

**Methods:**

This qualitative study used a phenomenological research design and was conducted from July 2019 to January 2021. A semi-structured topic guide was developed based on literature and the Common-Sense Model of Self-Regulation. We conducted one focus group discussion and 15 in-depth interviews with primary care HCWs. Interviewees were 7 physicians and 11 allied HCWs who tested positive for LTBI by Tuberculin Skin Test or Interferon Gamma Release Assay. Audio recordings were transcribed verbatim and thematic analysis was used to analyse the data.

**Results:**

We found four factors that serve as barriers to HCWs’ LTBI treatment uptake. Uncertainties about the need for LTBI treatment, alongside several other factors including the attitude of the treating physician towards treatment, time constraints during clinical consultations, and concerns about the treatment itself. On the other hand, facilitators for LTBI treatment uptake can be grouped into two themes: diagnostic modalities and improving knowledge of LTBI treatment.

**Conclusions:**

Improving HCWs’ knowledge and informative clinical consultation on LTBI and its treatment benefit, aided with a definitive diagnostic test can facilitate treatment uptake. Additionally, there is a need to improve infection control measures at the workplace to protect HCWs. Utilizing behavioural insights can help modify risk perception among HCWs and promote treatment uptake.

## Introduction

Healthcare workers (HCWs) have an increased risk of active and latent tuberculosis infection (LTBI) compared to the general population [1–3]. In low- and middle-income countries (LMICs) where there is high tuberculosis (TB) incidence, a systematic review of 26 LMICs found that the prevalence and incidence of LTBI detected by the Tuberculin Sensitivity Test (TST) or Interferon Gamma Release Assay (IGRA) among HCWs are high [4]. A positive TST was detected in nearly 50% of HCWs, whereas 39% had a positive IGRA. LMICs with an annual TB incidence of less than 300 per 100 000 had the highest prevalence of LTBI, with more than half testing positive for TST or IGRA [4]. In Malaysia a country with an intermediate TB burden, the HCWs are at twice the risk of contracting TB although with a lower mortality rate compared to the general population [5]. These findings indicate increased workplace exposure to mycobacterium tuberculosis despite existing guidelines on the prevention of TB among HCWs [6]. A study conducted in a tertiary hospital in Malaysia using TST showed a prevalence of 50% among HCWs, which was comparatively higher than a past study among a similar population using IGRA which showed a prevalence of LTBI of only 10.6% [7, 8].

Various recommendations for treating HCWs with LTBI have resulted in uncertainty and a lack of treatment uptake among HCWs in Malaysia [9]. Recently, the Asian Latent Tuberculosis (ALTER) expert panel advocates treatment of LTBI among HCWs in countries with intermediate and high TB burden, which has prompted the Ministry of Health Malaysia to actively screen and treat HCWs with a 6-months Isoniazid regime starting from September 2020 [9]. However, in 2021 there were updates to the guideline which now includes the option of 3- months treatment regime of Isoniazid and Rifampicin in addition to the existing regime [10]. Despite this, the acceptance and completion of LTBI treatment among HCWs remains unsatisfactory. Barriers identified were medication related side effects, long duration of treatment (6 to 9 months), frequent follow-up visits and some of them stopped treatment without any specific reasons [11–13]. To date, existing data on facilitators and barriers on LTBI treatment among primary healthcare workers in Malaysia is lacking. Therefore, the objective of this paper is to examine the facilitators and barriers to LTBI treatment among primary healthcare workers to provide a better understanding on this matter in order to improve treatment uptake and assist policymakers plan for strategies.

## Methods

This qualitative study used a phenomenological research design and was conducted from July 2019 to January 2021. Participants were purposively selected from primary HCW with current or history of LBTI (positive TST or IGRA and negative chest X-ray finding). Participants were recruited from 6 primary healthcare clinics in Petaling district, Selangor, Malaysia. A semi-structured interview guide was developed based on the Common-Sense Model of Self-Regulation (CSM-SR) and literature review [14]. The topic guide consists of open-ended questions covering topics related to possible factors influencing decision to receive or not receive LTBI treatment.

### Data collection

Researchers approached the medical officers in charge of the occupational and safety unit of the primary care clinics to identify HCWs with a diagnosis of LTBI. These identified HCWs were then approached if they were interested to participate in this study. HCWs who agreed were given an appointment for an interview at the convenience of participants.

In the preparatory phase, a pilot Focus Group Discussion (FGD) was conducted with 4 HCWs of different job categories to assess the suitability of the topic guide and method. The pilot FGD lasted about two hours and IDIs lasted about one hour. On average, all interviews were conducted for one hour. Although no sensitive or significant issues emerged, some participants were influenced by the response of other participants. Therefore, subsequent interviews were carried out using In-depth Interview (IDI). Findings from the pilot interview were included in the data analysis.

Interviews were conducted face-to-face in a private consultation room within the primary care clinics by two experienced qualitative researchers (SNFH and AM). The interviews were conducted in English and Malay language (national language of Malaysia) at participants’ preferences. Ethical approval to use face to face interview method had been obtained, however due to the COVID-19 pandemic and nationwide Movement Control Order (MCO) during the later date of data collection, several IDIs were conducted through telephone during the MCO (with the additional approval from the ethics committee).

### Data analysis

Interviews were audio recorded and transcribed verbatim and checked for accuracy. Transcripts in the Malay language were analysed in the original language by researchers who are fluent in both English and Malay languages. Coding and analysis were done using the both languages as well, and were conducted independently by WMK, AM, and SNFH with the use of NVivo version 12. Thematic Analysis by Braun and Clarke was used to analysed the data [15]. An initial coding framework was formed after discussion among the researchers and with other team member. The remaining transcripts were coded using the coding framework and any discrepancies were resolved by agreement within the research team. Emerging themes were formed. Non-English quotes were subsequently translated by a certified translator and checked for accuracy by the researchers.

### Reflexivity

SNFH is a researcher who conducted interviews for all categories of HCWs. Since her background is in health education and promotion across all disease categories, she was able to maintain a high degree of impartiality during data collection and analysis. AM is a primary care physician and conducted interviews only among allied HCWs, identifying herself as a researcher. AM, WMK and EMK had constantly reminded themselves of the potential influence of their job as primary care physicians during the data analysis.

### Data saturation

Data saturation was achieved on the sixteenth interview, and an additional two interviews were done to confirm data saturation.

## Results

**Fig. 1:**
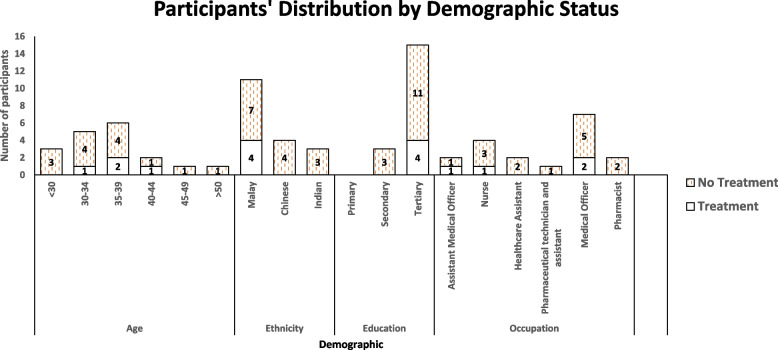
Participants’ distribution by demographic status

A total of 15 face-to-face interviews and three audio phone interviews were conducted among the HCWs. In these six primary healthcare clinics, 18 HCW were diagnosed with LTBI. 16 were diagnosed through TST, and two were diagnosed with IGRA. Seven medical officers (doctors), four nurses, two pharmacists, one pharmaceutical technician and assistant, two assistant medical officers and two health care assistants were approach and all agreed to be interviewed. Out of 18, Four participants had accepted treatment and 14 refused. Figure 1 summarises the sociodemographic profile of the participants.

### Barriers for LTBI treatment uptake

**Fig. 2:**
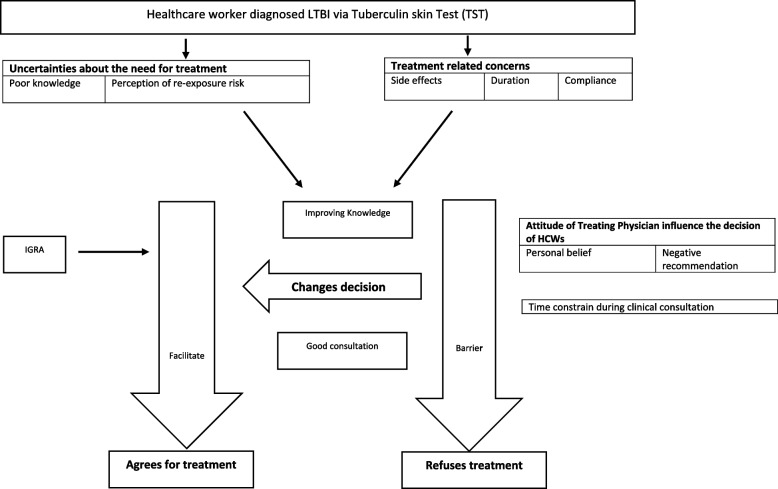
Barriers and facilitators influencing HCW’s decision-making to accept or refuse LTBI treatment

This study found the four barriers for HCW LTBI treatment uptake. The uncertainties about the need for LTBI treatment was found to be barrier to accept the treatment, alongside several other barriers including the attitude of treating physician influenced treatment, time constraints during clinical consultation and treatment related concerns. These factors are described in more detail under the respective four themes below, and summarised in Fig. 2.

### Theme 1: uncertainties about the need for LTBI treatment

There were two subthemes which contributed to LTBI treatment refusal. These uncertainties occurred due to poor knowledge on LTBI and its treatment benefit, and TB re-exposure risk at the workplace.

#### Poor knowledge on LTBI and its treatment benefit

One of the factors contributing to the refusal of LTBI treatment is the poor knowledge among healthcare workers (HCWs) regarding LTBI and its treatment benefits. HCWs who lack understanding about LTBI may not perceive the severity of the infection, leading them to refuse the recommended treatment.

*"My decision to not take the treatment at that point of time because I actually do not have the knowledge. I don’t know is it important to treat latent tb, as how it is important to treat tb…I perceive it is not serious."*

(P12, 36 years old, refused treatment, nurse)

One HCW who perceived low severity of LTBI compared to active TB and other diseases, resulting in deprioritizing LTBI treatment.

*"I know it is wrong to want to wait until it becomes active (TB) before I want to take the treatment. Now I don’t feel the thing (LTBI treatment) is a priority that I have to be treated for that. Maybe I’m more worried about high cholesterol, high sugar, once I think it is necessary… but as for now, I don’t think it’s a priority for me to treat my latent TB."*

(P8, 52 years old, refused treatment, doctor)

#### Perception of re-exposure risk at the work place

Uncertainties on LTBI treatment also occurred due to HCWs repeated exposure risk at the workplace. HCWs felt that there was no need to treat LTBI as they would be continuously exposed to TB infection due to their work nature and environment and did not want to undergo repeated treatment courses.

*“Because for me, every day we are facing patients with TB, so once you are exposed to TB patients, you are very high risk. … So, let say if I decided for six months (LTBI treatment) and then suddenly I get active TB, so I have to take another six months (treatment). So, if I take the medication there is no guarantee I will not get it some day later”.*

(P1, 36 years old, refused treatment, assistant medical officer)

### Theme 2: attitude of the treating physician influenced the treatment

The attitude of the treating physician plays a significant role in influencing HCWs treatment uptake. Attitude of treating physician who include personal belief and negative recommendation influenced HCW to refuse LTBI treatment uptake. When HCWs receive negative recommendations from their treating physicians, it can reinforce their reservations and uncertainties about the treatment. The authority and expertise of the physician carries significant weight, leading the HCW to prioritize the physician’s opinion.

*"I asked for his opinion (doctor). If you are in my position, would you take it? He said no, he will not take (treatment). I did ask him why he would not take (treatment) because he said we healthcare workers are exposed to that thing (TB) moreover there is no symptom (decided not to take)."*

(P12, 27 years old, refused treatment, nurse)

### Theme 3: time constraints during clinical consultation

Due to time constraints during clinical consultations, there is a lack of knowledge transfer between the treating physician and HCWs, which resulted in HCWs not receiving the knowledge they require to make decisions.

*"Because at that time the doctor wanted to hurry. So the way he said… I don’t quite get it (understand). I mean I don’t quite understand what is said. If the doctor emphasized (the importance of treatment), if he explained in more detail, maybe I will also think “oh okay can take (treatment)."*

(P12, 27 years old, refused treatment, nurse)

Lack of time during consultation leads to the treating physicians not providing detailed explanation on the importance of LTBI, resulting in these HCWs failing to perceive the importance of the treatment. A detailed explanation of LTBI is necessary in assisting HCWs in their decision-making process.

*"I would like to see someone (doctor) who can explain to me regarding the treatment in more detail. And if it’s okay, I don’t mind. If it’s counselled properly, I don’t think I mind taking the medication."*

(P13, 33 years old, refused treatment, doctor)

### Theme 4: treatment related concerns (side effect, duration and compliance) side effect

#### Side effect

Participants stated that treatment side effects were a barrier to treatment uptake. This included the side effects of treatment in general, and also specifically on the perceived harm of the side effects to babies who are being breastfed by the HCW during the treatment period.

*"That day according to him (doctor) I really should take(treatment) but I am the one that reject, not wanting to take because I am breastfeeding. But the doctor said I can take treatment even though I am breastfeeding but to me, my baby is still small at that time, so I am worried la afraid of side effect to him(baby) taking medicine, its TB medicine right, the dose is high also, right. So, I said is it possible for me to hold off first, not to take any medicine now la."*

(P16, 31 years old, refused treatment, nurse)

*"Yes, working in pharmacy, every medication has its side effect."*

(P18, 49 years old, refused treatment, Assistant Pharmaceutical Officer)

#### Duration

The 6-months treatment duration was considered a factor hindering HCWs from initiating the treatment. Long treatment duration was mentioned as a reason for hesitating or refusing to undergo the treatment. HCW felt overwhelmed by the idea of committing to a six-month course of medication, which can create doubts about their ability to complete the course of treatment successfully.

*"Also because of thought of having to take the medications for six months ( little laugh ) the durations la."*

(P18, refused treatment, doctor)

#### Compliance

The requirement for daily adherence to medication posed a challenge for HCWs considering LTBI treatment. Some HCWs expressed concerns about their ability to comply with the treatment regimen, including the fear of forgetting to take medication daily. HCWs also raised concerns about acquiring drug-resistant strains due to non-compliance.

*“I have to adhere to it (treatment)…To take medication every day, I am afraid I will forget. Not talking about other people. I’m talking about myself”.*

(P8, 52 years old, refused treatment, doctor)

*"I worry about my compliance. I have no habit of taking medication every day… so I worry if I start, then I will not be compliant. Then it will become if I ever get reactivation or whatever, I may get resistant strain."*

(P14, 34 years old, refused treatment, doctor)

#### Facilitators for LTBI treatment uptake

The facilitators for LTBI treatment uptake can be grouped into three themes. The first theme is diagnostic modalities followed by the second theme; improving knowledge on LTBI treatment.

### Theme 1: diagnostic modalities

Diagnostic modalities play a crucial role in influencing treatment decisions for LTBI infection among healthcare workers (HCWs). The availability of confirmatory diagnostic tests, such as Interferon-Gamma Release Assay (IGRA), can significantly impact the willingness of HCWs to undergo LTBI treatment. HCWs value the availability of reliable and confirmatory diagnostic modalities for LTBI. The use of IGRA, which provides more accurate results compared to traditional methods like the TST, enhances HCWs’ confidence in the diagnosis and facilitates their decision to undergo treatment.

*"So, from the IGRA test, I think I am confident la that… before, we just had Mantoux (test), chest X-ray but it was not confirmatory that you really have the infection (LTBI). So, from IGRA test is positive and I think I need to take the treatment. So, I took (treatment)."*

(P7, 43 years old, accept treatment, doctor)

*“I assume that the technique (TST) was wrong….To confirm that it’s really the latent TB or is it because of the technique (TST), (I would do another test, and if it is positive)…I will consider to take the medicine.*

(P11, 35 years old, refused treatment, pharmacist)

### Theme 2: improving knowledge on LTBI treatment

Improving knowledge on LTBI treatment and its benefits plays a crucial role in changing treatment decisions and facilitating treatment uptake among healthcare workers (HCWs). Access to educational courses or clinical consultations can significantly contribute to expanding HCWs’ understanding of LTBI treatment.

*"No one explained the benefit of taking (treatment) before this. No one pushed (for treatment) … I went for a course at that time. Indeed, at that time it was eye-opening too. He (from the course) said it is better I receive the treatment… Even though I am exposed to the risk continuously, but I think by taking it (treatment), it can reduce somehow the risk of infection compared to not taking treatment at all."*

(P7, 43 years old, accept treatment, doctor)

*"I would like to see someone (doctor) who can explain to me regarding the treatment in more detail. And if it’s okay, I don’t mind. If it’s counselled properly, I don’t think I mind taking the medication."*

(P13, 33 years old, refused treatment, doctor)

HCWs who have knowledge on the benefit of LTBI treatment were aware about the risk of active TB in the future due to their work nature. Consequently, they willingly choose to undergo the treatment.

*"Why I decide (to take the treatment)? Number one is I want to be on the safe side, because in the near future, I may still be seeing some TB or potential TB patients. Number two, for me… extra benefit to reduce the risk of active TB so why not?"*

(P6, 32 years old, accept treatment, doctor)

## Discussion

Findings from this study showed that themes were interrelated and influenced HCWs’ decision to accept or refuse LTBI treatment. Uncertainties on the need for LTBI treatment and treatment-related concerns, when coupled with a poor consultation; where the attitude of the treating doctor, and time constraints in the consultation serve as a treatment barrier. A definitive diagnostic test such as IGRA and providing HCWs with knowledge on the LTBI and its treatment benefit can result in treatment uptake and in some instance a change of decision from refusal to acceptance.

Uncertainties about the need for LTBI treatment and poor knowledge on LTBI leads to the underestimation of the severity and seriousness of LTBI. HCW viewed other diseases as priority as compared to LTBI and failed to perceive the severity of active TB. Similar findings were seen in many preventive medicine strategy in particular influenza or COVID vaccination among HCW where vaccination was viewed as a potential harm by HCW rather than a step taken for disease prevention [16, 17]. To address these uncertainties first we need to address the gap in knowledge of LTBI among HCW. In this study, HCW choose to take treatment for active TB over a preventive treatment for LTBI focusing on similar duration, and forsaking other issues of active TB such as infectivity and health complications [10]. This was made worse from the attitude of the treating physicians, where the treating physicians personal believes were included when providing consultations. Limited consultation duration, where concerns and understanding of the HCWs on LTBI and its treatment benefit were not addressed adequately further lead to treatment refusal. These findings were similar to other studies where there is a knowledge gap on LTBI among HCWs on top of a lack of decision making competency when it comes to own treatment decisions [16, 18, 19]. These important knowledge gaps were not addressed by the treating physicians due to various factors. One of the reason could be due to insufficient evidence and the limited capacity of existing evidence to address inquiries in real-world scenarios [20]. To address this, in addition to continuous medical education, a Latent Tuberculosis Decision Aid that incorporates personalised estimates for the risk of tuberculosis (TB) reactivation, TB death, quality-of-life impairments, and treatment side effects will be able to facilitate and increase treatment initiation and uptake among treating physician and HCWs [21]. To improve initial assessment and treatment initiation, continuous medical education on current recommendations and guidelines and digital solutions should be provided to HCWs and treating physicians [22]. Dedicated LTBI clinic with well-trained physicians on providing LTBI care with follow up clinic visits providing adequate time for HCW to make decision and follow up on treatment related concerns could further enhance decision uptake [13]. Similar to vaccination strategy, one of the key elements leading to HCW acceptance to LTBI treatment is the through education, debunking myths and eliminating fear in particular the fear of treatment side effects [17].

LTBI treatment concerns were one of the main barriers to treatment for LTBI. Treatment concerns includes side effects of the drugs, long duration of treatment and compliances [23]. Risk analysis models often disregards patients’ inconvenience which plays an important role in treatment decisions [24]. Inconvenience found in this study were the long treatment duration of six months isoniazid therapy and compliance concerns as a result of HCWs’ erratic working schedules. Shorter treatment duration and single pill combinations are associated with a more positive treatment uptake as it improves compliance and eliminates the fear of developing resistance as a result of non-compliances [25–27].

Ultimately, we found that treatment decision for LTBI were influenced by the knowledge and understanding of LTBI. HCW with adequate knowledge on LTBI decides to take treatment. Uncertainties on treatment benefits impedes decisions to proceed with the treatment [18, 19, 28, 29]. due to a lack of awareness and knowledge of LTBI treatment [30, 31]. Through education and knowledge transfers, treatment decision can be changed and treatment uptake improves [32–34].

Providing a definitive diagnostic test such as the IGRA testing to HCW with positive TST could enhance treatment uptake. Some HCWs felt that TST was not diagnostic and there is a possibility of false positive results due to multiple factors such as the misconception of history of BCG vaccination and working in an environment with continuous exposure to active TB [35]. In TST-positive patients, studies had supported the use of IGRA to reduce false positive results of TST and improve clinical management of LTBI [36, 37]. A study showed that two thirds of HCW were discharge from LTBI clinic without treatment as the treating physician regards a positive TST as false positive reflecting a previous BCG vaccination and HCW were at a low risk of reactivation [38]. The use of IGRA could address this issue and provide aids in convincing treatment uptake as it is a better predictor of latent TB infection [39, 40]. However, the role of serial IGRA testing among HCWs who are diagnosed with LTBI but refuse treatment is currently not done given the lack of data on the optimum cut offs for serial testing and unclear interpretation and prognosis of conversions and reversions, studies recommend a good correlation between a positive IGRA and occupational risk like working in high risk setting and duration of service [41–43].

In Malaysia studies on the prevalence LTBI among HCWs were mainly done in tertiary hospitals and there is scarcity of data on the prevalence of LTBI in the community or primary healthcare. Currently, the Ministry of Health Malaysia’s strategy of screening for LTBI among HCWS includes a mandatory TST and chest x-ray screening at the time of employment followed by screening of HCWs working at high risk setting areas every three years or upon exposure to an active TB case. According to The Programmatic Management of Latent Tuberculosis Infection, the risk factors for LTBI among healthcare workers includes: working in clinical areas, duration of employment more than five years, aged ≥ 35, close contacts, having chronic disease, working as a nurse and being male [44]. Based on our findings, this study proposes that personalised counselling should be given based on each HCWs’ risk factors.

To reduce the risk of TB among HCWs, LTBI treatment alone may not be adequate, a national surveillance program coordinated by Occupational, Safety and Health Department with annual reminder on TB symptoms to HCWs should be considered [45]. To reduce exposure risks at primary healthcare facilities, infrastructure modification to ensure appropriate natural ventilation/airflow and provision of personal protective measures should be considered. To reduce Tuberculosis infection in HCWs, monitoring and reporting of HCWs who develop TB and LTBI should be done annually at all primary healthcare facilities [4]. HCWs who tested positive who refuse treatment, annual TB symptom screening and chest X-Ray should be done among HCWs and should be promptly evaluated should they develop symptoms suggestive of TB [46]. Usage of face mask should be emphasised among HCWs especially when managing patients suspected or diagnosed with TB as evidence shows a significant reduction in TB risk [47].

### Strengths and limitation

This study has several limitations. Since all of the participants were primary care HCWs in the Petaling district, Selangor, their views may differ from HCWs from other districts and states in Malaysia. They also underwent clinical training in primary care in different institutions and received different levels of on-the-job training, and may reflect different values when compared to HCWs trained in other clinical specialties. To address this, future research should include a wider variety of HCWs.

The major strength of this study is that it reveals specific factors related to the perceptions of HCWs in determining their decisions to initiate treatment. Our study suggests that the appropriate level of awareness and knowledge on LTBI treatment among HCWs is an important determinant. Therefore, focused on strengthening effective training and clinical audits may be critical in increasing treatment uptake among HCWs.

## Conclusion

The study conducted sheds light on the decision-making process of healthcare workers (HCWs) when it comes to the treatment of LTBI. It reveals that HCWs who possess good knowledge and understanding of the importance of LTBI treatment are more likely to perceive the severity of the condition, leading to a higher likelihood of treatment uptake. However, the availability of a definitive investigation can further facilitate this decision-making process.

Conversely, uncertainties surrounding the necessity of treatment, stemming from inadequate knowledge and perceived risk of re-exposure, along with concerns about treatment side effects, duration, and compliance, contribute to treatment refusal among HCWs. Based on the findings of the study, it is recommended to offer Interferon-Gamma Release Assay (IGRA) testing to HCWs as a definitive diagnostic tool to assist them in their decision-making process. IGRA testing provides more accurate and reliable results, thereby enabling HCWs to make informed decisions regarding LTBI treatment.

Utilizing behavioural insights can be valuable in modifying the risk perception among HCWs and promoting treatment uptake. By understanding the factors that influence HCWs’ decision-making, MOH can design interventions that address specific barriers and misconceptions, ultimately encouraging HCWs to prioritize LTBI treatment.

## Data Availability

The datasets collected and analysed for this study are not made publicly available since data consists of interview transcripts, and the need to maintain anonymity of the participants. Additional data required can be requested through the corresponding author.

## Abbreviations

ALTER: Asian Latent Tuberculosis expert panel
COVID-19: Coronavirus disease 2019
CSM-SR: Common-Sense Model of Self-Regulation
FGD: Focus group discussion
HCW: Healthcare workers
IDI: In-depth interview
IGRA: Interferon Gamma Release Assay
LMICs: Low- and middle-income countries
LTBI: Latent tuberculosis infection
MCO: Movement control order
TB: Tuberculosis
TST: Tuberculin skin test
